# Kyste irien post traumatique chez l'enfant

**DOI:** 10.11604/pamj.2014.17.194.4024

**Published:** 2014-03-13

**Authors:** Ryme Abdelkhalek, Said Chetoui

**Affiliations:** 1 Service d'Ophtalmologie de l'Hôpital Militaire d'Instruction Mohamed V Rabat, Maroc

**Keywords:** Kyste, iris, traumatisme, oeil, cyst, iris, trauma, eye

## Image en medicine

Les kystes de l'iris sont rares chez l'enfant. Ils peuvent être primitifs ou secondaires, congénitaux ou acquis, pigmentés ou non pigmentés. L’échographie à haute fréquence précise le diagnostic et oriente la thérapeutique. Ces tumeurs bénignes posent le problème de leur extension locale et de leur caractère récidivant. Nous rapportant le cas d'un garçon de 9 ans qui consulte pour rougeur oculaire gauche avec photophobie évoluant depuis une semaine. L'interrogatoire trouve la notion de traumatisme contusif il y a un an. L'examen ophtalmologique révèle une acuité visuelle de 5/10, un kyste irien clair avec quelques pigments sur sa surface à base inféro-nasale, des milieux transparents et l'examen de l’oeil droit était normal avec une acuité visuelle à 10/10. Les kystes iriens chez les enfants sont rares. Il est important de les dépister et de les traiter précocement afin d’éviter le risque d'amblyopie. Le diagnostic est rendu facile grâce à l'ultra-biomicroscopie. Le meilleur traitement reste encore à définir.

**Figure 1:**
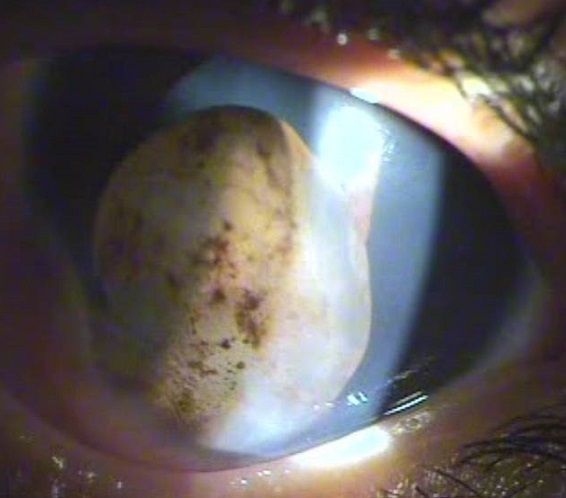
Kyste irien clair pigmenté

